# Soil acidification and the importance of liming agricultural soils with particular reference to the United Kingdom

**DOI:** 10.1111/sum.12270

**Published:** 2016-06-24

**Authors:** K. W. T. Goulding

**Affiliations:** ^1^ Department of Sustainable Soils and Grassland SystemsRothamsted ResearchHarpendenAL5 2JQUK

**Keywords:** Acid deposition, fertilizer, liming, lime requirement, soil acidification

## Abstract

Soil acidification is caused by a number of factors including acidic precipitation and the deposition from the atmosphere of acidifying gases or particles, such as sulphur dioxide, ammonia and nitric acid. The most important causes of soil acidification on agricultural land, however, are the application of ammonium‐based fertilizers and urea, elemental S fertilizer and the growth of legumes. Acidification causes the loss of base cations, an increase in aluminium saturation and a decline in crop yields; severe acidification can cause nonreversible clay mineral dissolution and a reduction in cation exchange capacity, accompanied by structural deterioration. Soil acidity is ameliorated by applying lime or other acid‐neutralizing materials. ‘Liming’ also reduces N_2_O emissions, but this is more than offset by CO_2_ emissions from the lime as it neutralizes acidity. Because crop plants vary in their tolerance to acidity and plant nutrients have different optimal pH ranges, target soil pH values in the UK are set at 6.5 (5.8 in peaty soils) for cropped land and 6.0 (5.3 in peaty soils) for grassland. Agricultural lime products can be sold as ‘EC Fertiliser Liming Materials’ but, although vital for soil quality and agricultural production, liming tends to be strongly influenced by the economics of farming. Consequently, much less lime is being applied in the UK than required, and many arable and grassland soils are below optimum pH.

## Introduction

The pH of agricultural soils is almost always measured in water, although 0.01m calcium chloride is sometimes used for research purposes (e.g. Blake *et al*., 1999) because it simulates the soil solution better than water. UK agricultural soils usually have a pH in water of between 5 (unlimed mineral soils) and 7.5 (chalky or limestone soils). Peats can have a pH of <4 and, if the mineral soils beneath them contain pyrite and are oxidized when the peat is removed, they can attain a pH of 2. Sodic (sodium saturated soils, e.g. from sea water ingress) can have a pH >8.

Lime was used by the Romans 2000 years ago to offset ‘sourness’ (i.e. acidity) on agricultural land and its use has been practised for centuries (Goulding *et al*., 1989; Connor *et al*., 2011). The basic elements of soil acidity and liming do not change: a useful and comprehensive description of it can be found in Adams (1984), Kennedy (1992) and Rengel (2003). This study briefly sets out the basics of soil acidification and then reviews the current (2016) UK status of acid deposition, soil pH, lime use, its impact on carbon (C) sequestration and greenhouse gas emissions and the continuing need for lime.

### Soil acidification

The acidification of soil is caused by:

1. acidic precipitation in its true sense, that is H^+^ ions in precipitation;
2. the deposition from the atmosphere of acidifying gases or particles such as sulphur dioxide (SO_2_), ammonia (NH_3_) and nitric and hydrochloric acids (HNO_3_; HCl);
3. the application of acidifying fertilizers such as elemental sulphur (S), urea or ammonium ($\text{NH}{}_{4}^{+}$) salts and the growth of legumes such as clover;
4. nutrient uptake by crops and root exudates;
5. the mineralization of organic matter.

### Acidic precipitation

‘Pure’ rain is usually slightly acid, with a pH of between 5 and 5.6 because of the dissolution of carbon dioxide (CO_2_) and the dissociation of the resulting carbonic acid (H_2_CO_3_). A soil exposed to such rain, but no other acidifying inputs and receiving no lime, would attain the same equilibrium pH as that of the rain. There are, however, very strong localized effects because human activity has increased the acidity of precipitation through emissions of acidifying compounds such as SO_2_ and nitrogen oxides (NOx) from industry and motor vehicles, and NH_3_ volatilized from manures and fertilizers (RoTAP, 2012).

### Acidifying gases and particles

From the beginnings of the Industrial Revolution until the 1970s, S emissions increased and SO_2_ was the main component of acid deposition (RoTAP, 2012). However, by the 1990s, S deposition had decreased to a fraction of what it was 30 years earlier because of the decline in heavy industry and the switch from coal to natural gas as an energy source: data for Woburn Farm, Bedfordshire, showed a decline in total S deposition (sulphate, ${\text{SO}}_{4}^{2-}$, in precipitation plus SO_2_) from approximately 85 kg/ha/year in 1970 to approximately 15 kg/ha/year in 1995 (McGrath & Zhao, 1995). The current total S deposition at Woburn is <5 kg/ha/year (RoTAP, 2012) and is likely to decline even further towards approximately 2.5 kg/ha/year by 2030 (S. P. McGrath, personal communication). Nitrogen (N) deposition had become the dominant pollutant in acid rain in 1998, much of which was acidifying. It remains dominant, but at Rothamsted its deposition has decreased from a peak of 40–50 kg/ha/year to arable land in the early 1990s (Goulding *et al*., 1998) to approximately 25 kg/ha/year today. Much of this decline has been caused by the fitting of catalytic converters to vehicles and a general reduction in emissions from combustion (RoTAP, 2012). Current acid deposition rates are equivalent to no more than 2 kmol H^+^/ha/year.

### Acidifying fertilizers and legumes

The most important causes of soil acidification on agricultural land are the application of ammonium‐based fertilizers and urea, elemental S fertilizer and the growth of legumes (Bolan & Hedley, 2003). Ammonium salts strongly acidify soils through the process of nitrification

(1) $$\text{NH}{}_{4}^{+}+2O_{2}=\text{NO}{}_{3}^{-}+2H^{+}+H{}_{2}O$$

**Figure 1:**
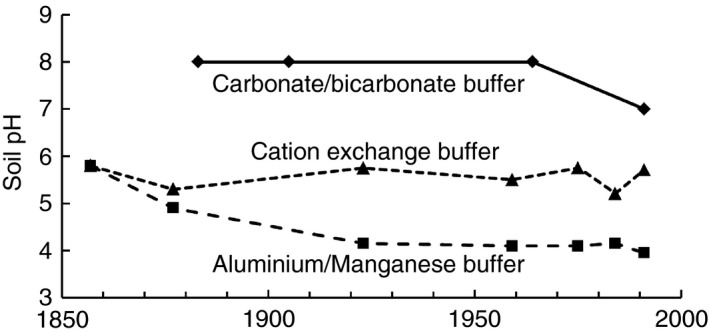
Examples of three of the buffering mechanisms in soils: carbonate/bicarbonate buffer in the soil under Broadbalk Wilderness, where acid deposition has been buffered by very large quantities of chalk applied in the 19th century; cation exchange buffer in the Park Grass unlimed ‘Nil’ treatment (no fertilizer or manure applied so acidification only from acid deposition); aluminium/manganese buffer in the Park Grass unlimed, ammonium sulphate fertilizer treatment that has experienced severe acidification.

If the nitrate $\left(\text{NO}{}_{3}^{-}\right)$ is taken up by the crop, there is no net acidification because the $\text{NO}{}_{3}^{-}$ takes up protons with it (Marschner, 2012; 14.4). Acidification only occurs when $\text{NH}{}_{4}^{+}$ is nitrified and the $\text{NO}{}_{3}^{-}$ leached. The same is true of urea: there is no net acidification if all the N in the urea is utilized by the crop; acidification only occurs when the urea is converted to $\text{NH}{}_{4}^{+}$ , the $\text{NH}{}_{4}^{+}$ nitrified and the $\text{NO}{}_{3}^{-}$ leached. Ammonium sulphate applied to some plots of the Park Grass Experiment at Rothamsted has caused a rapid decrease in pH, starting in the surface soil (Figure 1) but occurring throughout the profile to at least 1 m. The larger the application rate the more rapid the rate of acidification (Johnston *et al*., 1986).

The decline in S deposition noted above has resulted in the need for farmers to apply S fertilizer as explained in the Fertiliser Manual (RB209; Defra, 2010). Inputs of S as elemental S or as SO_2_ from the atmosphere produce acidity when they are oxidized, that is

(2) $$2S+3O_{2}+2H{}_{2}O=2H{}_{2}\text{SO}{}_{4}$$

(3) $$2{\text{SO}}_{2}+O_{2}+2H{}_{2}O=2H{}_{2}\text{SO}{}_{4}$$

but ${\text{SO}}_{4}^{2-}$ produces no acidity because it is not subject to further oxidation.

The fixation of atmospheric N_2_ by legumes results in the formation of $\text{NH}{}_{4}^{+}$ within the root nodules by nitrogenase, the uptake of an excess of cations, especially K^+^, and therefore a net release of protons to balance the charge (Marschner, 2012; Ch 14.4). Bolan & Hedley (2003) reported that, where legumes had been grown continuously in Australia for >30 years, soil pH had declined by 1 unit.

### Nutrient uptake by crops and root exudates

Plant growth and nutrient uptake result in some localized acidification around plant roots through the exudation of acids from the roots (Hinsinger *et al*., 2003). Excluding the particular case of legumes, the contribution of this to bulk soil acidification is small (<10%) when compared with N and S fertilizer inputs (Johnston *et al*., 1986) but it has an important influence on the bioavailability of plant nutrients in the rhizosphere (Marschner, 2012).

### Mineralization

When microorganisms decompose soil organic matter they produce CO_2_, which dissolves in soil water to form H_2_CO_3_ in the same way as in rain. Thus, soil and root respiration can result in a large concentration of CO_2_ in soil air, but because acidic soil solutions hold very little CO_2_, the process is unlikely to cause soil pH to decline below 5 (Bolan *et al*., 2003).

## Impacts of soil acidification

### Effects on soils

Soils are ‘buffered’ against acidification by a series of chemical processes: (i) firstly, the dissolution of carbonates and other basic rocks, the ‘carbonate/bicarbonate’ buffer, then (ii) the replacement of exchangeable base cations [calcium (Ca^2+^), magnesium (Mg^2+^), potassium (K^+^) and sodium (Na^+^)] by H^+^ and aluminium (Al^3+^) through the cation exchange (CEC) buffer and then (iii) the dissolution of Al‐bearing and manganese minerals, if manganiferous minerals proliferate, and finally (iv) the dissolution of iron‐bearing minerals. These processes buffer the pH at approximately 7–8, 5–6, 4 and 3, respectively. Thus soil acidification results in periods of constant or slowly decreasing pH while one process buffers inputs, followed by a relatively rapid decrease in pH when that process is exhausted and the next takes over. Examples of field soil buffer curves from the Long‐Term Experiments at Rothamsted can be seen in Figure 1.

**Figure 2:**
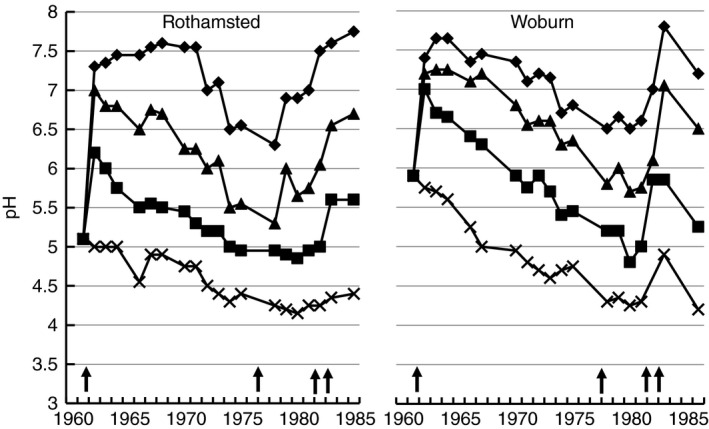
Soil pH measured in samples taken from experimental treatments of the Long‐Term Liming experiments at Rothamsted and Woburn. Key: Rothamsted: 0 (X), 5 (■), 10 (▲) and 20 (♦) t/ha lime; (b) Woburn: 0 (X), 4.6 (■), 10.9 (▲) and 17.3 (♦) t/ha lime. The vertical arrows (↑) point to years when lime was applied.

There are no observable effects of acidification on soil while lime or chalk buffers the system (e.g. Figure 1); there is merely a large loss of CO_2_ and Ca^2+^ (or Mg^2+^ in dolomitic limestone areas). Once cation exchange becomes the main buffer, essential nutrient cations (Ca^2+^, K^+^, Mg^2+^) are leached, base saturation decreases together with nutrient availability, Al^3+^ saturation increases and crop yields begin to decrease. Figure 2 shows the changes in soil pH on treatments of the Long‐Term Liming experiments on the silty clay loam soil at Rothamsted and the sandy loam soil at Woburn, where there was no free lime and the soils were in the base cation buffer range. With no lime applied, but with growing crops receiving fertilizer, the pH declined continually at both sites, faster on the sandy loam soil at Woburn with its smaller clay content and, therefore, smaller base cation buffer capacity.

On entering the Al/Mn and Fe buffer ranges, significant, nonreversible changes to the soil begin that involve clay mineral dissolution and a reduction in cation exchange capacity (CEC), accompanied by structural deterioration. Such weathering is not reversible except over geological timescales and so represents a serious and costly degradation of soil quality (Blake *et al*., 1994). Soil acidification, if not corrected, can extend deep into the subsoil, as on the Park Grass and Geescroft Wilderness Experiments at Rothamsted (Blake *et al*., 1999). Such extreme acidification will take a long time and considerable expense to rectify.

### Effects on crop plants

Crop plants vary in their tolerance to acidity. Table 1 (adapted from MAFF (Ministry of Agriculture, Fisheries and Food), 1981) summarizes the sensitivity of the more common crop plants and forage species to soil pH; an extensive list of minimum soil pH values for arable crops, grasses and clovers, vegetables and fruit can be found in MAFF (Ministry of Agriculture, Fisheries and Food) (1981). The critical soil pH also varies with soil texture and crop cultivar and so critical values quoted in the literature vary. Many US state extension services provide tables of critical and recommended soil pH values such as Oregon (east of the Cascades: Horneck *et al*., 2006; western Oregon: Anderson *et al*., 2013) and Washington (Froese *et al*., 2015). Comparing the US tables with Table 1 shows variations of usually 0.1‐0.3 pH units, for example from Horneck *et al*. (2006), 6.5 for alfalfa (lucerne) cf 6.2 in Table 1, and 6.0 for red clover cf 5.9 in Table 1. Other authors and extension services quote a range of critical pH values, for example 5.5‐6.0 for wheat and 6.0–6.5 for Alfalfa (Fageria *et al*., 1997); CSIRO in Australia provides a comprehensive table of critical pH ranges for crops, pastures and fruit (Hazelton & Murphy, 2007). However, as it is not possible to manage soils to obtain the whole range of crop‐specific pH values in a crop rotation, the advice for the UK (Defra 2010) and most other countries is to maintain soil pH values at optimal values of 6.5 (5.8 in peaty soils) for cropped land and 6.0 (5.3 in peaty soils) for grassland.

**Table 1:** Soil pH values below which crop growth may be restricted on mineral soils (adapted from MAFF (Ministry of Agriculture, Fisheries and Food), 1981, Appendix 2)

Crop	Critical soil pH	Forages	Critical soil pH
Field bean (*Vicia faba*)	6.0	Lucerne (*Medicago sativa*)	6.2
Barley (*Hordeum vulgare*)	5.9	Vetch (*Vicia sativa*)	5.9
Sugar beet (*Beta vulgaris*)	5.9	Red clover (*Trifolium spp*.)	5.9
Pea (*Pisum sativum*)	5.9	White clover (*Trifolium spp*.)	5.6
Oilseed rape (Canola; *Brassica napus*)	5.6	Timothy (*Phleum pratense*)	5.3
Maize (*Zea mays*)	5.5	Cocksfoot (*Dactylis*)	5.3
Wheat (*Triticum aestivum*)	5.5	Rye (*Secale cereal*)	4.9
Kale (*Brassica oleracea var. acephala*)	5.4	Fescue (*Festuca*)	4.7
Swede (*Brassica napus var. napobrassica*)	5.4		
Linseed (*Linum usitatissimum*)	5.4		
Turnips (*Brassica rapa*)	5.4		
Oat (*Avena spp*.)	5.3		
Potato (*Solamum tuberosum*)	4.9		

The pH of the soil affects the bioavailability of plant nutrients and so, indirectly, crop plant growth. The well‐known but important optimum pH values for a range of plant nutrients are shown in Table 2.

**Table 2:** Optimum soil pH values for the availability of the major and the most important micronutrients (adapted from Foth, 1990)

N	P	K & S	Ca & Mg	Fe	Mn	B, Cu & Zn	Mo
6–8	6.5–7.5	>6	7–8.5	<6	5–6.5	5–7	>7

Other than chalky or limestone soils, agricultural soils will require applications of lime periodically to neutralize the acidity caused by crop and livestock production, whatever the farming system. The study now addresses how to calculate the ‘lime requirement’ and the materials that can be used for liming.

## Lime requirement

Liming has been considered in the context of replacing Ca^2+^ leached as the balancing cation with bicarbonate (HCO_3_^−^), chloride (Cl^−^), $\text{NO}{}_{3}^{-}$ and ${\text{SO}}_{4}^{2-}$, and Ca^2+^ removed in farm products (e.g. Bolton, 1977; Johnston & Whinham, 1980). Thus, theoretically from Equation [1] and considering only nitrate leaching,

(4) $${\text{Ca}}^{2+}+2\text{NO}{}_{3}^{-}=\text{Ca}{\left({\text{NO}}_{3}\right)}_{2}$$

Some 3.6 kg CaCO_3_ is required to balance the Ca^2+^ lost with $\text{NO}{}_{3}^{-}$ when 1 kg ammonium‐N is nitrified; at field rates, this is approximately 180 kg CaCO_3_ for every 50 kg ammonium‐N. There are situations in which it is appropriate to use Ca^2+^ to displace exchangeable Al^3+^ and raise base saturation rather than aiming to increase pH (Adams, 1984). However, for increasing soil pH, the most appropriate method is to calculate the lime needed to neutralize the acidity produced in by each ammonium ion, which generates two protons when nitrified (Eq. (1)) (Adams, 1984; Johnston *et al*., 1986; Kennedy, 1992):

(5) $${\text{CaCO}}_{3}+2H^{+}={\text{Ca}}^{2+}+{\text{CO}}_{2}+H{}_{2}O$$

This produces double the lime requirement to that calculated for Ca^2+^ loss, that is the nitrification of 50 kg ammonium‐N requires 360 kg CaCO_3_ to neutralize the acid formed. It more closely matches field observations of the lime requirement than the amount needed to replace leached cations and reflects the increasing amount of lime required to correct soil acidity as the pH decreases. The pH scale is logarithmic; soil at pH 5 theoretically has ten times the H^+^ ion activity than that soil at pH 6. Thus, theoretically, the lime requirement does not increase linearly as pH declines. On the Park Grass Experiment at Rothamsted, <5 t lime/ha will increase soil pH from about 6–7 over 5 years, but nearer 20 t/ha are needed to increase it from 5 to 7. The lime requirement also varies with soil texture and organic matter content or, more correctly, the buffering capacity (BC) of the soil. Thus, for the same required change in pH, soils with a low BC, such as sands, require less lime than those with a higher BC, such as clays.

### Measuring the lime requirement

For many years, in England and Wales, the standard method for measuring the lime required to adjust the pH of a soil was the buffer method of Woodruff (1948). Soil pH was measured after equilibration with a calcium acetate/p‐nitrophenol/magnesium oxide buffer and the lime requirement was calculated by applying a factor to the difference between the measured and target pH. From 2000, the Fertiliser Recommendations (Defra 2000) and now the Fertiliser Manual (Defra, 2010) have used look‐up tables (Table 3) based on the analysis of many years’ data by ADAS and others that showed a linear relationship between soil pH and lime requirement, with a slightly different relationship for each soil textural class. Lime recommendations for Scottish soils are based on a very similar table (Sinclair *et al*., 2014).

**Table 3:** Lime recommendation tables as used in the Fertiliser Manual (RB209; Defra 2010)

Initial soil pH	Sands and loamy sands		Sandy loams and silt loams		Clay loams and clays		Organic soils		Peaty soils	
	Arable	Grass	Arable	Grass	Arable	Grass	Arable	Grass	Arable	Grass
	Lime (t/ha)									
6.2	3	0	4	0	4	0	0	0	0	0
6.0	4	0	5	0	6	0	4	0	0	0
5.5	7	3	8	4	10	4	9	3	8	0
5.0	10	5	12	6	14	7	14	7	16	6

Sands = all sands and loamy sands; light = all sandy loams, sandy silt loam, silt loam; medium and clay = all clay loams and clays; organic = 10–25% organic matter; peat and peats = >25% organic matter. ‘Arable’ refers to a soil depth of 20 cm and ‘grassland’ to a soil depth of 15 cm.

Lime requirement calculators have been available for many years. Rothamsted constructed a lime requirement model, RothLime ( http://www.rothamsted.ac.uk/rothlime; Goulding *et al*., 1989) based on data from the Long‐term Liming experiments at Rothamsted and Woburn and the Park Grass Experiment. The lime requirement was calculated from measured changes in pH with time following lime applications, as in Figure 2. RothLime considers soil type, crop (arable/grass), the neutralizing value (NV) of product to be used and acid deposition on a regional basis. It has a wide range of original and target pH values (4.5–7.0) but, like most other recommendation systems, does not incorporate the effects of acidifying fertilizers or legumes. The Agricultural Lime Association (ALA: http://www.aglime.org.uk/lime_calculator.php_calculator.php) also has an online Lime Calculator. Recommendations from these calculators correspond well to those in the Fertiliser Manual (Defra, 2010).

These methods calculate the lime needed to correct soil acidity once it has been caused. It is possible to estimate the amounts of lime needed to counteract soil acidification caused by acid deposition, acidifying fertilizers and legumes, and so avoid rather than correct acidity. Such estimates are, however, very variable because acidification from these inputs depends on the weather, soil type, management system and especially the efficiency of use of any N and S inputs. Estimates from the literature (e.g. Bolan & Hedley, 2003; Upjohn *et al*., 2005) give the following:

1. acid deposition equivalent to 25 kg ammonium‐N/ha/year requires approximately 250 kg CaCO_3_/ha/year;
2. 50 kg/ha/year ammonium‐N fertilizer requires approximately 360 kg CaCO_3_/ha/year, but estimates vary from 200–500 kg CaCO_3_/ha/year;
3. 50 kg/ha/year urea‐N fertilizer requires approximately 100 kg CaCO_3_/ha/year, but poor efficiency of use could increase this to 200 kg CaCO_3_/ha/year;
4. 30 kg/ha/year elemental S require 94 kg CaCO_3_/ha/year based on equations [3] and [5], but adjusting this to a ‘field rate’ increases the amount to approximately 150 kg CaCO_3_/ha/year; if sulphate is used as the source of S there will be no acidification;
5. intensively managed, legume‐based dairy pastures fixing 250 kg N/ha/year and ammonium‐nitrate‐based grazing systems receiving the same amount of N require approximately 400 kg CaCO_3_/ha/year, but poor efficiency of N use could increase this to 1000–1200 kg CaCO_3_/ha/year.

## Liming materials

### Most commonly used materials

The most commonly used liming materials are ground limestone, dolomitic ground limestone, chalk, ground chalk, burnt lime and hydrated lime; almost 70% of the material currently used in the UK is ground limestone. Their definitions or ‘Meaning’, specifications and ‘Declarations’ (what the buyer must be told about them) must comply with the Fertiliser Regulations 1991 (GB Statutory Instruments, 1990). For example, ‘ground limestone’ means ‘sedimentary rock consisting largely of calcium carbonate and containing not more than 15% of magnesium expressed as MgO and of which 100% will pass through a sieve of 5 mm, not less than 95% will pass through a sieve of 3.35 mm and not less than 40% will pass through a 150‐micron (150 *μ*m) sieve’. The seller must also declare the neutralizing value (NV) and the amount of material as a percentage by weight that will pass through a 150‐micron sieve. The NV of the material defines the amount of acidity that it will neutralize and is based on a reaction with HCl in a laboratory. Typical NVs of the three most commonly used materials are as follows:

1. Limestone (CaCO_3_), NV = 50–55% depending on the geological strata;
2. Dolomitic limestone (CaMg(CO_3_)_2_, usually 42% CaCO_3_ and 53% MgCO_3_), NV = 56%;
3. Chalk (CaCO_3_), more readily broken down and absorbed into the soil solution than limestone, NV = 48–54%.

However, the effectiveness of a liming material also depends on its reactivity, effectively its rate of dissolution, which depends on particle size and hardness. For example, the difference between ‘ground’ and ‘screened’ limestone is the amount that will pass through a 150‐micron sieve: not less than 40% of the former and not less than 20% of the latter; that is screened limestone is a coarser material and so it reacts more slowly.

Throughout Europe, each country has its own specifications for liming materials but the European Union has proposed harmonizing regulations. EC Regulation 463/2013 adds liming materials to the European Fertiliser Regulations so that they can be sold as ‘EC Fertiliser Liming Materials’, in which case sales documentation must state the parent rock type (e.g. Chalk), the grade of product, the NV and the Ca^2+^ and/or Mg^2+^ content.

### Other acid‐neutralizing materials

A number of ‘waste products’ are available that neutralize acidity: sugar factory lime, basic slag, wood ash, coal combustion products such as fly ash and bottom ash, calcium humates and fulvates from oxidized brown coal and by‐products of the paper and pulp industry (e.g. Bolan *et al*., 2003; Gagnon *et al*., 2014). The NVs of some of these, compared with lime‐based products, are shown in Table 4. Sugar Factory (or Spent) Lime is a by‐product of sugar beet purification. It also contains some nutrients, approximately 3–5 kg N, 7–10 kg ‘available’ P_2_O_5_, 5–7 kg MgO and 4–6 kg SO_3_ per tonne of lime and has a fine particle size, so is fast‐acting.

**Table 4:** The neutralizing value of various liming materials expressed as a weight percentage of pure lime (CaCO_3_) adapted from Bolan *et al*. (2003)

Liming material	Chemical formula	Neutralising value
Burnt lime	CaO	179.0
Slaked lime	Ca(OH)_2_	136.0
Dolomitic lime	CaMg(CO_3_)_2_	109.0
*Lime*	*CaCO*_*3*_	*100.0*
Basic slag	CaSiO_3_	86.0
Phosphogypsum	CaSO_4_.2H_2_O	0.3
Mined gypsum	CaSO_4_.2H_2_O	12.4
Flue gas desulphurised gypsum	CaSO_4_.2H_2_O	0.1
Coal fly ash		Variable

Italicised text shows lime as the reference against which other acid neutralising materials are compared.

Gypsum and phosphogypsum have small NVs. They are most effective in soils rich in variable charge components, such as Fe and Al oxides, in which some acidity is neutralized by the OH^−^ ions released during the adsorption onto the oxides of ${\text{SO}}_{4}^{2-}$ from the gypsum and phosphogypsum. This is sometimes referred to as the ‘self‐liming effect’ (Bolan *et al*., 2003).

Phosphate rock can have a liming value of between 450 and 560 kg CaCO_3_ equivalent per tonne applied due to the presence of some CaCO_3_ and the dissolution of the mineral, which consumes H^+^ (Bolan *et al*., 2003). Paper waste can have a liming value of between 0.1 and 0.7 pH units rise per 100 t/ha of waste applied (Gibbs *et al*., 2005).

### Precision or Variable Rate Liming with pelletized lime

Pelletized lime consists of aggregates of 2–5 mm diameter comprising finely ground and/or micronized particles of CaCO_3_ or MgCO_3_ held together with a cementing agent that facilitates storage, transportation and application but dissolves when the granules are applied to moist soils. It is usually specified as having at least 90% of the aggregated particles passing a 150‐micron sieve. The cost of pelletizing the lime makes it considerably more expensive than ground limestone, so some see it as a maintenance material applied in smaller amounts than bulk lime (Higgins *et al*., 2012). With this approach, when the soil pH is considerably below the optimum, ground limestone would be applied, followed by an annual application of pelletized lime when the required pH is reached. Comparing pelletized lime and ground limestone at the same rates from the same source, Higgins *et al*. (2012) found no difference: both maintained or slightly increased the soil pH, particularly in the top 2.5 cm of the profile.

**Figure 3:**
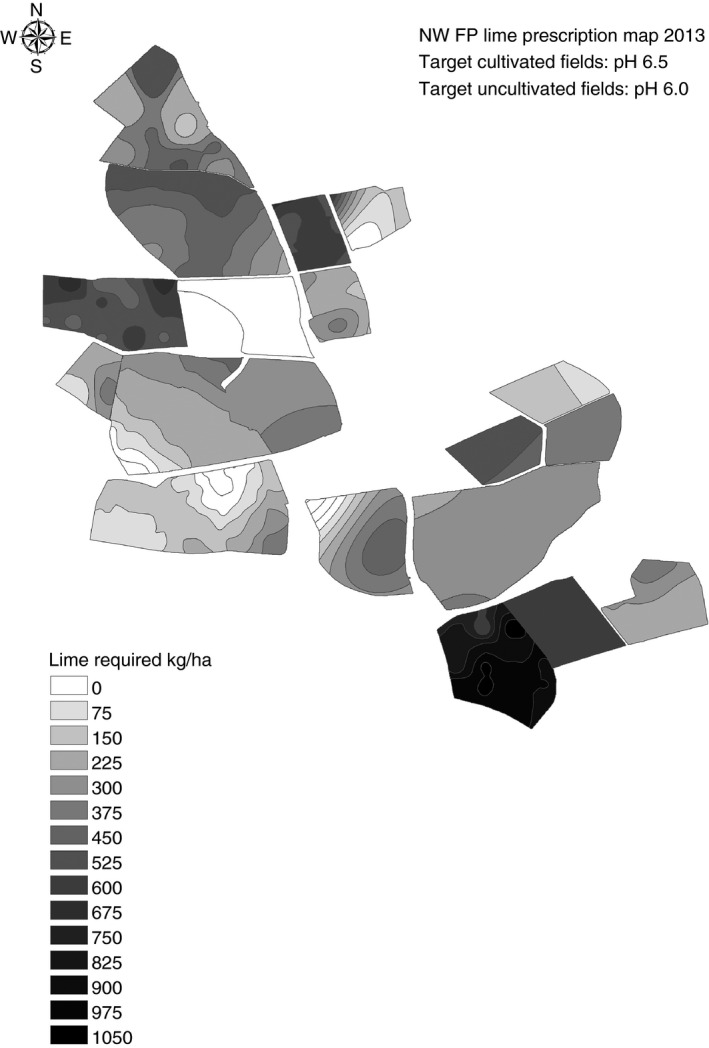
Variable‐rate lime recommenations for fields of the ‘North Wyke Farm Platform’, a farm scale experiment at North Wyke, Devon, UK, at which basic operations and measurements are made and other experiments superimposed. (Robert Orr, private Communication).

Variable rate application (VRA) of pelletized lime is being applied to the fields of the North Wyke Farm Platform (a series of experimental fields or ‘farmlets’ at North Wyke in Devon, UK, on which three contrasting livestock production systems are being compared; Orr *et al*., 2016) at rates of up to 1 t/ha (Figure 3). Of the four fields treated to date with initial pH values of 5.5–5.8, recommended applications have achieved the target pH of 6.0 in the field with an initial pH of 5.8 but not in the other three fields, falling short by 0.2 units.

## Effects of liming on soils, productivity and biodiversity

Liming increases Ca^2+^ concentrations and ionic strength in the soil solution, causing clay flocculation and thus an improvement in soil structure and hydraulic conductivity (Haynes & Naidu, 1998). Liming also increases earthworm activity and therefore macroporosity (Bolan *et al*., 2003). Because of the beneficial influence of lime on soil structure, there has been much research on the use of lime and other acid‐neutralizing materials for improving degraded soils, especially in arid and semi‐arid countries, for example Kirkham *et al*. (2007). Bennett *et al*. (2014) found that lime applied at 5 t/ha was still improving aggregate stability, hydraulic conductivity, vegetation cover, total C and N and soil respiration 12 years after application.

Dolomitic limestone is recommended for soils deficient in Mg^2+^ but using it too frequently can result in Mg^2+^ indices >3 and so poor K^+^ availability. In such a situation, farmers should ensure that there is sufficient K^+^ available and so no risk of K^+^ deficiency in the crops grown.

Farmers and agronomists are well aware of the importance of lime for maintaining soil quality and crop yields in arable soils, but grassland areas tend to be neglected, especially when the economics of livestock production are poor. Johnston *et al*. (2001) showed the complex interaction between lime and nutrients in determining productivity and species richness in grassland. Anderson (2004) provided a specific example of the benefits to productivity of liming upland grassland soils using research that began in the 1970s: livestock numbers doubled within 4 years of lime application and clover persisted for over 20 years. However, there is a conflict in that liming has variable effects on biodiversity (Kirkham *et al*., 2008). Yu *et al*. (2010) examined the problems of reconciling productivity and biodiversity in Welsh upland pastures where lime had been applied in the early 1990s but not again until 2007. Acidification between lime applications caused an increase in mosses, dead grass and species regarded as agricultural weeds, and a reduction in stock carrying capacity and productivity, but some of the ‘agricultural weeds’ are of potential environmental benefit, for example to pollinators and birds. Morgan *et al*. (2008) looked at the most effective ways of restoring species‐rich, semi‐natural grassland in upland Wales and found that having the correct hay cutting and/or grazing management was most important, but that liming to pH 6 with the correct hay or grazing management produced more desirable species than in the same treatments without lime.

## Liming, carbon sequestration and climate change

One area of greatly increased interest in recent years has been the impacts of lime and liming on C sequestration by soils and thus on climate change. Paradelo *et al*. (2015) reviewed the literature and found that, on balance, liming increased soil C content mostly because it increased crop yields and therefore residue returns. Fornara *et al*. (2011) used data spanning 129 years of the Park Grass Experiment at Rothamsted to show that net C sequestration measured in the 0–23 cm layer at different time intervals since 1876 was 2–20 times greater in limed than in unlimed soils: the greater biological activity in limed soils, despite increasing soil respiration rates, led to plant C inputs being processed and incorporated into resistant soil organo‐mineral pools more effectively. They therefore concluded that liming might be an effective mitigation strategy against climate change. However, this has to be balanced against the emissions of CO_2_ when lime neutralizes acidity in soils (Equation (5)), which must be reported in national greenhouse gas inventories (De Klein, *et al*., 2006). Gibbons *et al*. (2014) looked at the trade‐off between lime applications and greenhouse gas (GHG) emissions on livestock farms. They found that liming to pH 6, as recommended in the Fertiliser Manual (Defra, 2010), reduced N_2_O emissions (and nitrate leaching) but, in CO_2_‐C equivalents, the GHG emissions from liming were about four times those saved by reducing N_2_O emissions.

## Lime use and the current pH status of UK soils

**Figure 4:**
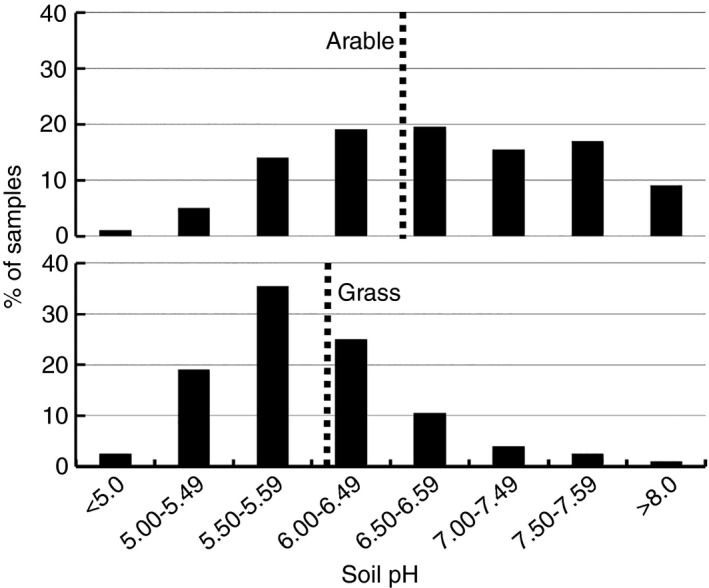
The pH status of >200 000 UK arable and grassland soils as measured by the Professional Agriculture Analysis Group in the 2013/14 season (PAAG, 2014). The dashed vertical line marks the soil pH recommended in the Fertiliser Manual (RB209; Defra, 2010).

Liming to the pH recommended in the Fertiliser Manual (Defra, 2010) is essential for good soil management, crop growth, nutrient use efficiency and environmental protection. The Professional Agricultural Analysis Group (PAAG, 2014) publishes an annual report summarizing the results of >200 000 annual soil analyses that includes a breakdown by pH class; results for the 2013/14 season are shown in Figure 4. Although not a statistically representative sample of UK soils, the data suggest that c 40% of UK arable soils are below the level recommended in the Fertiliser Manual of pH 6.5 and 57% of grassland soils are below the recommended pH of 6.

Data compiled from the Agricultural Lime Producers Council, the Ministry of Agriculture, Fisheries and Food to 2001 and the Department for Environment, Food and Rural Affairs thereafter and the British Survey of Fertiliser Practice show that insufficient lime is being applied: annual amounts applied have declined from c 6000–7000 kt product/year to c 2500 kt product/year today. This is much less than the calculated average annual lime loss for the UK of 4 250 000 t CaCO_3_, estimated by Goulding & Annis (1998).

## Conclusions

Although a short‐term saving, reducing or omitting the application of lime to correct acidity risks significant economic loss through unachieved crop yield and wasted fertilizer, plus an increased risk of the pollution of water and air by N and P fertilizers. Liming to recommended soil pH values increases productivity, benefits soil structure, improves degraded soils and, when used with other appropriate management practices, can benefit grassland biodiversity. It also reduces some greenhouse gas emissions, but this has to be set against the emissions of CO_2_ when lime reacts with soil acidity. Despite the significant reduction in acidic atmospheric deposition in the UK, the acidification from it together with ammonium‐N and elemental S fertilizers, the use of legumes for N fixation and crop growth and nutrient removals will continue to require significant amounts of lime or other acid‐neutralizing materials. However, the economics of farming continues to override agronomy in decision making for liming, and areas limed and amounts applied are well below what is necessary for maintaining recommended soil pH values.
